# Morphological assessment of oocyte quality during assisted reproductive technology cycle

**DOI:** 10.5935/1518-0557.20240034

**Published:** 2024

**Authors:** Romualdo Sciorio, Luca Tramontano, Pier Francesco Greco, Ermanno Greco

**Affiliations:** 1 Fertility Medicine and Gynaecological Endocrinology Unit, Department Woman-Mother-Child, Lausanne University Hospital, Lausanne, Switzerland; 2 Department of Women, Infants and Adolescents, Division of Obstetrics, Geneva University Hospitals, Boulevard de la Cluse 30, 1211 Genève 14, Switzerland; 3 Villa Mafalda, Centre for Reproductive Medicine, Rome, Italy; 4 Department of Obstetrics and Gynecology, UniCamillus, International Medical University, Rome, Italy

**Keywords:** medically assisted reproduction, non-invasive assessment, oocyte morphology, oocyte quality, oocyte biomechanical features, pregnancy outcomes, healthy offspring

## Abstract

Following the advancement of medically assisted reproduction (MAR) technology, and the rationale to extend the culture to the blastocyst stage, performing elective single embryo transfer (eSET), gamete quality and assessment have acquired large relevance in ART. Embryo quality is strictly correlated with gametes quality and culture conditions. Oocyte maturity assessment is therefore imperative for fertilization and embryo evolution. Mature oocytes at the metaphase II stage result in a higher fertilization rate compared to immature oocytes. Indeed, oocyte morphology evaluation represents an important and challenging task that may serve as a valuable prognostic tool for future embryo development and implantation potential. Different grading systems have been reported to assess human embryos, however, in many cases, it is still a major challenge to select the single embryo to transfer with the highest implantation potential. Further, eSET has conferred a challenge to embryologists, who must try to enhance embryo culture and selection to provide an adequate success rate, whilst reducing the overall number of embryos transferred. Above the standard morphological assessment, there are several invasive or non-invasive approaches for embryo selection such as preimplantation genetic testing, time-lapse technology, proteomics and metabolomics, as well as oxygen utilization and analysis of oxidative stress in culture medium. This short review is not designed to be a comprehensive review of all possible features that may influence oocyte quality. It does give, however, a brief overview and describes the prognostic value of the morphological characteristics of human oocytes on their developmental capacity following ART treatments.

## INTRODUCTION

Over the last decades, MAR has remarkably changed, from an experimental procedure to mainstream medicine, which nowadays is responsible for the birth of almost 10 million babies worldwide (De Geyter *et al.*, 2020). Oocyte assessment represents a fundamental feature to describe embryo competency, in terms of blastocyst formation and implantation potential. It is probably one of the limiting factors in female fertility, playing a critical role from the egg collection, across the fertilization, and lately at the embryo development stage (Gilchrist *et al*., 2008). Certainly, IVF success is due to the combination of several procedures, and one of these, probably the most important is ovarian stimulation (OS). The procedure necessitates the use of exogenous gonadotropins to stimulate the women’s ovaries, to generate multiple oocytes that will be lately collected transvaginally (Ip *et al*., 2023; Pacchiarotti *et al*., 2016). In vivo, each month one oocyte ovulates, and the maturation occurs as the result of long and natural follicle growth and selection. Whereas *in vitro* the utilization of OS removes the natural selection of follicles and allows the maturation of oocytes that probably would never grow or ovulate. These follicles of compromised quality might produce oocyte not fully competent, and thus induce fertilization failure, even with the use of intracytoplasmic sperm injection (ICSI) or poor embryo development (Swain & Pool, 2008). The fertilization success depends not only on sperm penetration but is correlated with several additional factors of oocyte physiology, and the concern is that following OS, a percentage of collected oocytes have a reduced viability and are eventually destined for fertilization failure or generate an embryo with low implantation potential (Swain & Pool, 2008; Marteil *et al*., 2009). Oocyte maturation cannot be determined by only assessment and presence of the polar body (PB), which represents the nuclear maturation, but rather comprises the cytoplasmic maturation, including those features, which sometimes are not visible at the microscope observation. Those mechanisms in the oocyte cytoplasm are critical for the production and storage of carbohydrates, lipids, proteins, and the regulation of metabolic processes indispensable for oocyte competence to support normal fertilization and embryo development (Warzych & Lipinska, 2020; Richani *et al*., 2021). Importantly, the oocyte quality depends not only on the nuclear maturation and mitochondrial genome, but also on the environment provided around itself in the ovary during the folliculogenesis (Nagy *et al*., 2009; Patrizio *et al*., 2007), and later in the embryology laboratory, which tries to mimic the physiological in vivo environment. Therefore, the main goal of this narrative review is to describe the value of morphological features of human oocytes on their developmental capability. Since morphologic evaluation alone may be not enough to measure oocyte competence, here, will be examined oocyte features and analytical markers that may be needed to support more complete information about oocyte quality and further embryo development.

## FOLLICLE GROWTH AND OOCYTE MATURATION

**Figure 1:**
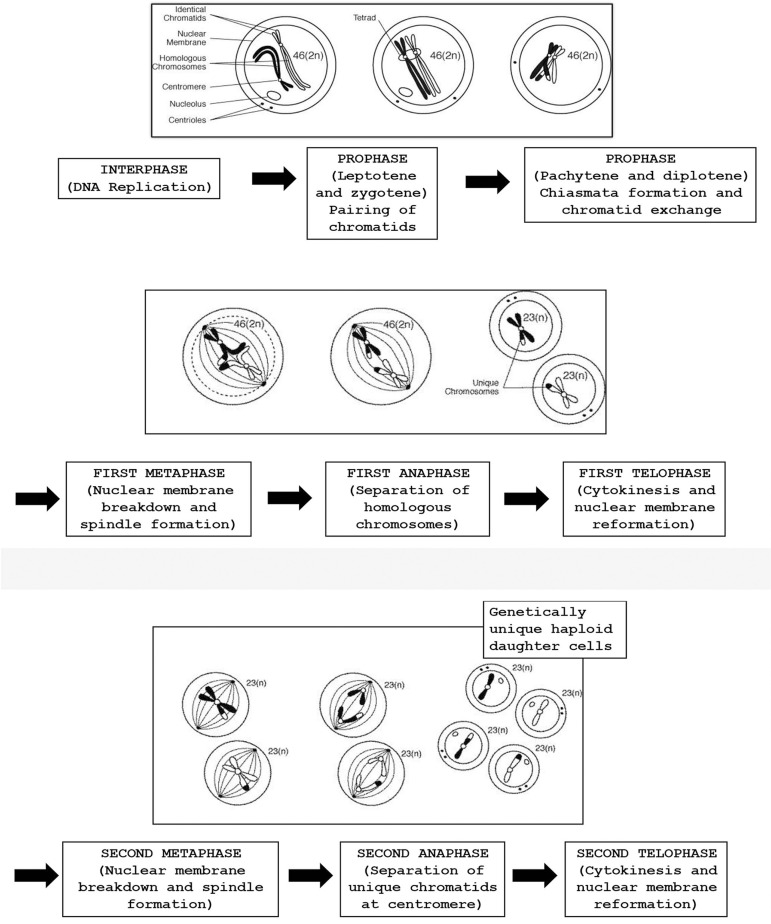
The process of meiosis with the formation of haploid gametes.

In MAR cycles with ICSI, the cumulus-oocyte complex (COC) before sperm injection needs to be removed. To clearly assess the oocyte nuclear maturation, the COC is placed in a medium containing a specific enzyme, the hyaluronidase, to detach its cells around, following mechanical force applied using a tiny sterile pipette. The observation by light microscopy of the first PB in the perivitelline space (PVS) is considered a marker of nuclear maturation. Oocytes with clear extrusion of the PBI are at the metaphase of meiosis II, with the chromatin aligned on the equatorial plate of the meiosis II metaphase spindle (Rienzi *et al*., 2003). This complex and articulate process of folliculogenesis begins very early during gestation: when primordial germ cells start to grow and migrate to the gonadal ridge, where they progress into oogonia. Between the third and fourth month of pregnancy, oogonia reaches the number of five or six million (Kerr *et al*., 2013; Kashi *et al*., 2023). They start meiosis and develop in primary follicles, which arrest at the prophase of meiosis I, which is commonly referred to as the germinal vesicle (GV) stage. The immature oocytes remain at this phase until puberty when they will be reactivated by circulating gonadotropins (Norris *et al*., 2009). Oocytes are allocated in the primordial follicles, which are formed by a single layer of flattened granulosa cells. Lately, some primordial follicles undergo growth and differentiation, with the conversion of the granulosa cells from flattened to cuboidal cells, to evolve into the primary follicles, and with further growth will form the secondary follicle (Sciorio *et al*., 2023; Barretta *et al*., 2023; McGee & Hsueh, 2000). At that time, the layer of granulosa cells expands and develops gap junctions and the receptors for follicle-stimulating hormone (FSH) (Albertini *et al*., 2001). Under the action of FSH, the small fluid-filled cavity will compose the antrum that provides nutrients to the oocyte. With the increase of the antrum cavity, follicles progress and form the tertiary follicles and lately the preovulatory follicles (Rodgers *et al*., 2001; Soyal *et al*., 2000). The preovulatory surge of luteinizing hormone (LH) triggers the meiosis I arrested oocyte to resume meiotic division and the formation of the mature COC, which encloses an oocyte arrested in the metaphase of meiosis II (MII) (Eppig, 1991; Guzeloglu-Kayisli *et al*., 2012). It is essential that MII oocytes contain a meiotic spindle (MS) to support regular chromosome alignment and segregation and avoid aneuploidies in the future embryo (Wang *et al*., 2001). Generally, following the OS it is expected that around 80-85% of the oocytes retrieved are at the MII stage, about 5-10% are at the GV stage and another 5-10% of oocytes with the absence of both PBI and GV are classified as being at MI. These oocytes have gone through GV breakdown but have not fully completed meiosis I and are still between MI and MII, where the chromosomes are aligned on the metaphase plate in preparation for finishing the first meiotic division (Tilia *et al*., 2020; Rienzi & Ubaldi, 2009). Figure 1 rates different stages of the meiosis process.

## CHROMOSOME SEGREGATION, RESUMPTION OF MEIOSIS, AND SPINDLE

At birth, primordial follicles enclosing oocytes quiescent at the prophase of the first meiotic division. During follicle development, it is reported an active growth phase, in which the oocyte achieves full size and then the follicle will be ready for ovulation. This stage is characterized by active RNA transcription. Thus, the oocyte DNA content needs to be dispersed and transcriptionally active to allow interaction with the transcriptional machinery. Once growth is achieved, the oocyte reaches the capability to restart meiosis and experiences a very active process of DNA compaction, which is transcriptionally inactive in preparation for the meiotic resumption (Coticchio *et al*., 2015; Tilia *et al*., 2020; Swain *et al*., 2008; Mattson & Albertini, 1990). The chemical compound cyclic adenosine monophosphate (cAMP) plays an important role in the regulation of meiotic arrest before ovulation (Edwards, 1965). The process of meiosis starts with the replication of the genetic material during the S-phase, followed by two successive chromosome divisions, with the results of a haploid chromosome constitution. The meiotic process provides the reduction of the chromosome numbers from diploid to the haploid set, typical of a gamete (Petronczki *et al*., 2003; Herbert *et al*., 2015). The spindle apparatus is a cytoskeletal structure that is actively involved in the correct chromosome segregation (Howe & FitzHarris, 2013; Inoué & Sato, 1967; Zhang & Galardy, 2016). Its fibers are formed by filaments called microtubules, which are dynamic structures able to dis-assemble and re-assemble since they are made of heterodimers of alpha and beta tubulin in association with microtubule-associated proteins (MAPS) (Montag *et al*., 2008; Moghadam *et al*., 2022). For a competent mature MII oocyte, the identification of the MS localized underneath the first polar body is mandatory (Montag *et al*., 2008). Several studies have examined the importance of MS in human oocytes, and its presence has been associated with fertilization rates and pregnancy outcomes with different results. Some studies reported that oocytes with MS showed significantly higher fertilization, pregnancy, and implantation rates (Tilia *et al*., 2020; Shen *et al*., 2006; Rienzi *et al*., 2005), whereas others (Chamayou *et al*., 2006; Fang *et al*., 2007) did not observe any significant difference. Collectively the stability and the presence of MS are associated with the healthy state of the mature MII oocyte. It is worth mentioning that the daily routine work in the embryology laboratory, and the culture conditions might affect the oocyte cytoskeleton, including damage to the MS, resulting in reduced fertilization rates (Taylor *et al*., 2008). It has been reported that MS stability might be impaired by suboptimal temperature. Indeed, human MS begins to depolymerize at a temperature of 33°C (Wang *et al*., 2002) and continues to depolymerize as temperatures drop, only about 5 to 10 minutes of exposure to non-physiologic temperature is sufficient to induce spindle disassembly (Montag *et al*., 2008). Further evidence on the adverse impact of *in-vitro* environmental variation including pH, temperature, and osmolality on MS has been established in both animal and human studies (Swearman *et al*., 2018). Overall, these studies showed that in the embryology laboratory is important to correctly monitor culture conditions and avoid variation, especially in pH and temperature to achieve an adequate fertilization rate and further embryo development (Swearman *et al*., 2018; Dal Canto *et al*., 2017; Pickering *et al*., 1990). Further, the MS dysfunction might be linked to errors in chromosomes division, and thus, accountable for aneuploidies. Unfortunately, it has been found that maternal age has a critical role in spindle formation: consequently, aberrations in this segregation process, especially during the first meiotic division in human eggs, can lead to an increased percentage of aneuploidies in embryos, and subsequent implantation failure or spontaneous abortion (Correa-de-Araujo & Yoon, 2021; Howe & FitzHarris, 2013). This has been the object of several published studies, which have confirmed similar results (Wasielak-Politowska & Kordowitzki, 2022; Blengini *et al*., 2021; Kuliev & Verlinsky, 2004; Ebrahimzadegan *et al*., 2023; Capalbo *et al*., 2013). A prospective longitudinal cohort study performed by Capalbo *et al*. (2013) found that in advanced maternal-age women (age > 40), almost 80% of gametes display abnormal spindles and chromosome alignment, compared to only 20% in younger patients aged 25 or under.

## OOCYTE COMPETENCE IN ART: CYTOPLASMIC MATURATION

Oocyte morphological assessment is routinely performed by the embryologist at the time of oocyte recovery under the microscope. It is considered non-invasive since is not interfere with the subsequent embryonic development and is compatible with the daily routine of busy IVF workflow. For a few decades, scientists have been trying to identify new features to successfully predict oocyte competence. Generally, it has been described as the association of two critical aspects: meiotic competence, which is the ability to resume and complete meiotic maturation, also identified as nuclear maturation, and cytoplasmic maturity, defined as the ability to generate a good quality embryo, capable of further development and results in a pregnancy to term (Xu & Zelinski, 2022). Female age, as mentioned earlier, is probably the most predictive feature of oocyte competence (Rienzi *et al*., 2011). However, a discrepancy exists between the competency of different oocytes collected from the same OS, and currently, the most reliable methods of determining oocyte competence are only marginally capable of predicting a successful pregnancy (Forman *et al*., 2013; Munné *et al*., 2019; Ozgur *et al*., 2019; Rienzi *et al*., 2011). Indeed, it is reasonable that as the capacity to anticipate oocyte competence continues to improve, OS procedures will be enhanced to select for quality over quantity of oocytes collected, to provide the patient treatment more friendly and cost-effective, reducing the proportion of ovarian hyperstimulation syndrome (Ciepiela *et al*., 2015). However, nuclear maturation alone is not sufficient for depicting oocyte quality, but it is also associated with the cytoplasmic maturity and mitochondrial genome, but also on the microhabitat supplied by the ovary and the preovulatory follicle, which influence both oocyte transcription and cytoplasmic maturity (Van Blerkom & Henry, 1992). In addition, oocytes carry mitochondria with their mitochondrial DNA, and in the future embryo, this is entirely provided by the maternal gamete (Shoubridge & Wai, 2007; Wai *et al*., 2010). Cytoplasmic assessment should be taken into consideration to establish ideal conditions for subsequent fertilization and embryo development. Mature oocytes should then incorporate a typical-clear looking cytoplasm, a smooth, and non-fragmented polar body, an adequate ZP thickness, and a small PVS (Swain & Pool, 2008). Regrettably, microscope evaluations are subjective and might diverge according to the operator’s experience and thus it is hard to have a validated predictive value in assessing the molecular signature of oocyte cytoplasmic maturation. These molecular mechanisms and signaling in the oocyte cytoplasm are essential for the production and storage of carbohydrates, proteins, RNAs, lipids, and fatty acids, successful organelles position and regulation of metabolic pathways required for oocyte maturation, competence to fertilization, and subsequent embryonic developmental capacity (Swain & Pool, 2008). Finally, multiple characteristics can change oocyte transcriptional activity and therefore impair the future embryo development potential of the future embryo. The entire range of factors that affect oocyte cytoplasmic maturation are yet to be identified, thus further studies need to completely clarify this aspect (Nagy *et al*., 2009; Patrizio *et al*., 2007).

## THE INFLUENCE OF NON-INVASIVE EVALUATION OF OOCYTE QUALITY

One of the most difficult challenges for the clinical embryologist is to select from a cohort of embryos the single one to transfer, applying the standard method of morphological assessment. It is well known that a considerable proportion of morphologically defined as good-quality embryos, still fail to implant, and produce a pregnancy, even after the application of the invasive and costly procedure of preimplantation genetic testing (Viotti, 2020). Surely, the goal of ART should be the delivery of a singleton healthy baby, and eSET should be necessary and routinely applied to avoid to the minimum the risk and difficulties correlated with multiple gestations (Tiitinen, 2019; Johnston *et al*., 2014). Therefore, the need to validate and adopt a non-invasive method to determine typical features of oocytes and embryos that mimic the normal health function or the ability to further develop into a healthy pregnancy is demanding. A novel approach, to be properly defined as non-invasive should be not harmful and not disturb the physiology of the oocyte, or capacity to be fertilized, to develop further to implant and result in a healthy baby. For this purpose, historically, morphologically microscopic evaluation has been applied to evaluate embryo viability. The observation at light microscopy of the oocyte’s cytoplasm has been the subject of many published trials that try to determine its association with fertilization and pregnancy outcomes. Some studies have investigated the oocyte cytoplasm and have defined the aspect as “dark cytoplasm” (Ten *et al*., 2007), while others found “dark granular appearance of the cytoplasm” (Esfandiari *et al*., 2006), “dispersed cytoplasmic granularity” (Rienzi *et al*., 2008), or “dark cytoplasm with granulation” (Balaban *et al*., 2008). Thus, some authors carefully examined this cytoplasm characteristic and tried to establish whether has an impact on pregnancy outcomes. It was reported that “dark cytoplasm” was not a predictive factor, thus it correlated neither with the fertilization rate nor with the embryo quality (Ten *et al*., 2007; Esfandiari *et al*., 2006). Whereas, other authors showed that embryo quality was compromised when embryos developed from oocytes with dark cytoplasm (Rienzi *et al*., 2008; Balaban *et al*., 2008). A trial performed by Wilding *et al*. (2007) showed that cytoplasmic granulation was associated with higher fertilization rates correlated to oocytes without any granularity. Therefore, the debate on dark and cytoplasmic granularity is still active; however, it is worth mentioning that these evaluations are very subjective and might have considerable discrepancy and variation among embryologists and laboratories.

## BIOMECHANICAL OOCYTE PARAMETERS

Recently it has been proposed by several authors the examination performed on the biomechanical properties of cells and their association with cell function and evolution. Viscoelasticity implies the property of materials that display elastic and viscous features when undergoing deformation. Cell membranes are an example of a viscoelastic material, as well as the intracellular cytoskeletal constitution and remodeling, which have correlated and impacted the cell’s viscoelastic nature (Evans & Hochmuth, 1976; Dimitrov, 1984; Gomez *et al*., 2021). In that line, the role of lipids, in the form of saturated and unsaturated fatty acids might alter the delicate metabolic balance necessary for the embryo to thrive (Gomez *et al*., 2021; Cortezzi *et al*., 2013; Haggarty *et al*., 2006; Yang *et al*., 2022; Shi & Sirard, 2022). Lipids and fatty are important for several biochemical processes required for embryo development, implicated in the arrangement of cellular phospholipid membranes for structural integrity to the provision of energy reserves for survival until successful implantation (Brusentsev *et al*., 2019). Several studies have demonstrated that interactive biophysical forces, such as cell membrane deformation and fluid-flow shear stresses, affect cellular viscoelasticity (Karcher *et al*., 2003; Shôji *et al*., 1978). In cancers as well as in stem cells, viscoelasticity has been shown to influence cell structure, function, and biophysical properties (Xu *et al*., 2012; Engler *et al*., 2006). Reproductive studies have currently proposed that assessment of oocyte biomechanical properties may contribute insight into the oocyte and subsequent embryo development. The cytoplasm viscosity (CV) of the oocyte has been the object of investigation by several authors, who have proposed that both viscosity and resistance have a significant effect on embryo development, in particular on fertilization, embryo quality, and blastocyst formation (Ebner *et al*., 2003). Further, it has been illustrated that during follicle maturation in OS regimes, the CV might experience ample modifications, going from more aqueous to more viscous and stickier. Generally, these novel findings declare that higher viscosity might be a poor prognostic indicator of embryo development and pregnancy outcome. Differences in CV have been also reported according to patients’ cause of infertility features and with ovarian stimulation protocols (Ebner *et al*., 2003; 2010). CV of mature oocyte and membrane resistance may be a critical aspect to take into consideration, and perhaps future studies will explain why some oocyte membranes break immediately at the ICSI time (and sometimes express degeneration soon after the sperm injection), while other oocytes fail to break even with aspiration (Yanez *et al*., 2016). Therefore, an additional relevant investigation should be performed on the zona pellucida (ZP), which is an oocyte-associated structure, and in the mammals, ZP is a sulfated glycoprotein matrix that is shaped enclosing the primary oocyte during the early stages of folliculogenesis (Rankin *et al*., 2000). The human ZP is formed by four glycoproteins (Lefièvre *et al*., 2004; Gupta, 2021; Gupta *et al*., 2012) and plays a crucial role at the fertilization time, during the binding of the spermatozoa to the oocyte (Albertini *et al*., 2001). It stimulates the acrosome reaction in the head of the spermatozoon (Ganguly *et al*., 2010) and soon after its binding and passage, the ZP structure changes and as such will avoid polyspermia (Wolf, 1981; Wassarman, 1999). Papi *et al*. (2010) using atomic force spectroscopy illustrated that the ZP of immature oocytes has a perfect elastic behavior, while in mature and fertilized oocytes, the ZP progresses to a more plastic behavior, and a higher force will be necessary to induce its deformation. However, it has been well known that after fertilization, the fusion of oocyte cortical granules and the discharge of their enzymatic contents into the perivitelline space results in ZP “hardening” and will represent an important mechanism to reduce the incidence of polyspermia (Wolf, 1981; Drobnis *et al*., 1988). A compelling study by Yanez *et al*. (2016) has performed a viscoelastic test on the mouse and human zygotes. The authors using a narrow micropipette aspiration platform that allowed quantification of the depth of aspiration of the ZP, and part of the cell membrane, managed to measure and quantify four biomechanical features of a linear elastic solid model for each zygote investigated. Despite these studies being encouraging and stimulating as potential non-invasive oocyte assessments, however, it is mandatory to perform powered prospective randomized controlled human studies to understand if these viscoelastic measures are predictive of developmental competence and pregnancy outcome (Yanez *et al*., 2016; Azarkh *et al*., 2023; Papi *et al*., 2010; Kort & Behr, 2017; Krause *et al*., 2016).

## POLAR BODY APPEARANCE

The polar body is located in the PVS and is typically smooth and without fragmentation. The biological and physiological relevance of PBI morphology, fragmentation, or dysmorphisms is currently obscure and still the object of big debate. The PBI fragmentation should not be addressed as an oocyte marker since the fragmentation may be associated with the post-ovulatory time. However, it has been proposed that a degenerated PBI might be correlated with asynchrony between nuclear and cytoplasmic maturation, probably due to the over-maturity of the oocytes (Eichenlaub-Ritter *et al.*, 1995). According to some authors, oocytes showing a clear and intact PBI without any fragment have a raised capacity to generate blastocysts and higher pregnancy rates (Ebner *et al.*, 1999; Mahfoudh *et al.*, 2017). However, studies have been conducted aiming to establish the relation between PBI morphology and ICSI outcome, but results did not show a neat link between the two characteristics (Fancsovits *et al.*, 2006). Further large PBI can be considered as a feature of poor prognosis and might be connected with compromised embryo viability, and to increased percentage of the embryo with multinucleated blastomeres and thus might support embryo aneuploidies (Fancsovits *et al.*, 2006; Mahfoudh *et al.*, 2017; De Santis *et al.*, 2005). Publishes studies seem to agree that most of the aneuploidies in the early stage of human embryos are carried from meiotic errors arising during oogenesis (De Santis *et al.*, 2005; Gorbsky, 2015; Nagaoka *et al.*, 2012). Recently, it has been proposed that chemical compounds and environmental pollutants, including endocrine disruptors, are depicting considerable warning to human reproductive health (Giudice, 2016; 2021; Segal & Giudice, 2022). On that line, preimplantation genetic testing for aneuploidies (PGT-A) has been enforced with the goal of determining euploid embryos to be replaced in IVF cycles (Handyside *et al.*, 1990). In particular, PB biopsy first intrroduced by Verlinsky *et al.* (1998) represents an alternative to day-3 or day-5 biopsy. An advantage of this application is the longer time available to perform genetic testing without the need to freeze the embryo; also, it avoids embryo manipulation, which might be critical in those countries where embryo manipulation is not allowed (Verlinsky *et al.*, 1998). However, the big disadvantage of the PB biopsy technique is that can discriminate only maternal aneuploidies and it cannot identify paternal meiotic or post-zygotic mitotic errors. Additional information on the application and results of PGT-A have been published by others (Verpoest *et al.*, 2018; Fiorentino *et al.*, 2014; Sciorio & Dattilo, 2020; Sciorio *et al.*, 2020; Handyside *et al.*, 1990).

## CONCLUDING REMARK

The combination of chemistry, microscopy, physics, genetics, genomics, and other biomedical technologies in the basic studies of oocyte biology and reproductive functions, represents the future of non-invasive assessment of oocytes. Novel analytical platforms for single oocyte evaluations are in expansion, including the assessment of oocyte transcriptomics (Reich *et al.*, 2011), single-cell polar body genomics (Hou *et al.*, 2013), mitochondrial genomics (Wolf *et al.*, 2015), and non-invasive genetic analysis of oocyte/embryo spent culture media (Huang *et al.*, 2019). These goals are not easy to achieve and fully validated to be applied routinely in the IVF laboratory, but improvement is being made. It is therefore important to support cross-discipline investigation to continue building a basement, and an accurate science that will provide a trustworthy biomarker of oocyte quality and developmental competence. Finally, main basic and applied science are both necessary in this adventure toward the establishment of non-invasive oocyte assessment.
